# Poisoning by Chlorate of Potash

**Published:** 1878-06

**Authors:** 


Poisoning by Chlorate of Potash.—A child of Dr. Kauffman, Schuylkill county, Pennsylvania playing doctor with her little brother and sister, found a box of chlorate of potash, of which, after administering various and unknown quantities to her patients, took about half an ounce herself. The child, who was about two and a half years old, commenced vomiting in about an hour, and died in seven hours after taking the medicine. Beside the emetic, also hydro-drastic effects were observed, the child dying of gastritis or inflammation of the stomach and bowels. This case teaches us to be very careful in using and keeping chlorate of potash. Under no circumstances whatever should it be administered in a crystalline form, as it seems to be in this form a violent irritant to the mucous membrane. The child was given as much water and cream as she could drink, and to the last she vomited crystals of chlorate of potash.—*American Journ. of Pharm.*

